# Cyclodextrins in Polymer-Based Active Food Packaging: A Fresh Look at Nontoxic, Biodegradable, and Sustainable Technology Trends

**DOI:** 10.3390/polym14010104

**Published:** 2021-12-28

**Authors:** Friné Velázquez-Contreras, Camilo Zamora-Ledezma, Iván López-González, Luis Meseguer-Olmo, Estrella Núñez-Delicado, José Antonio Gabaldón

**Affiliations:** 1 Molecular Recognition and Encapsulation Research Group (REM), Health Sciences Department, UCAM-Universidad Católica de Murcia, Campus de los Jerónimos 135, 30107 Murcia, Spain; fvelazqu@up.edu.mx (F.V.-C.); enunez@ucam.edu (E.N.-D.); 2 Escuela de Administración de Instituciones (ESDAI), Universidad Panamericana, Álvaro del Portillo 49, Ciudad Granja, Zapopan 45010, Mexico; 3 Tissue Regeneration and Repair Group Orthobiology, Biomaterials and Tissue Engineering, Health Sciences Department, UCAM-Universidad Católica de Murcia, Campus de los Jerónimos 135, 30107 Murcia, Spain; czamora9@ucam.edu (C.Z.-L.); ilopez27@ucam.edu (I.L.-G.); lmeseguer@ucam.edu (L.M.-O.)

**Keywords:** active packaging, biodegradable polymers, cyclodextrins, foods, shelf life

## Abstract

Using cyclodextrins (CDs) in packaging technologies helps volatile or bioactive molecules improve their solubility, to guarantee the homogeneous distribution of the complexed molecules, protecting them from volatilization, oxidation, and temperature fluctuations when they are associated with polymeric matrices. This technology is also suitable for the controlled release of active substances and allows the exploration of their association with biodegradable polymer targeting to reduce the negative environmental impacts of food packaging. Here, we present a fresh look at the current status of and future prospects regarding the different strategies used to associate cyclodextrins and their derivatives with polymeric matrices to fabricate sustainable and biodegradable active food packaging (AFP). Particular attention is paid to the materials and the fabrication technologies available to date. In addition, the use of cutting-edge strategies, including the trend of nanotechnologies in active food packaging, is emphasized. Furthermore, a critical view on the risks to human health and the associated updated legislation is provided. Some of the more representative patents and commercial products that currently use AFP are also listed. Finally, the current and future research challenges which must be addressed are discussed.

## 1. Introduction

Nowadays, most food packaging is used to guarantee food quality and safety by protecting it from external factors, including but not limited to odors, temperature, light exposure, and microorganisms [1]. Research and innovation in food packaging technologies have become an important part of the industry because they seek to improve safety and preserve the food’s organoleptic properties while maintaining product quality; they are also important due to the increased consumption of minimally processed foods, the demand for products without artificial preservatives, and the changes in food distribution practices associated with globalization [2,3,4]. As a result, food packaging is one of the most significant challenges in the food industry, particularly in the area of minimally processed foods, in which appropriate packaging helps to extend the food’s shelf life, avoiding microorganism invasion and deterioration. In this sense, the container, beyond fulfilling its basic functions of containing and protecting food, is evolving towards a multifunctional preservation receptacle that limits degradation while maintaining the food’s organoleptic properties and product quality [5].

**Figure 1:**
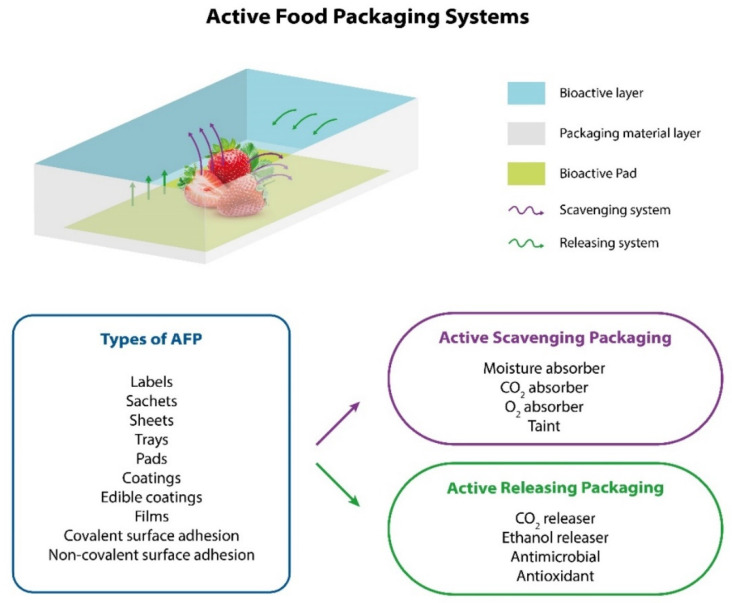
Schematization of the active food packaging (AFP) systems and their main features.

Active food packaging (AFP) plays an active role in preserving foods, extending their shelf life and improving their safety and sensory properties while maintaining food quality. They are classified into two main groups, active-scavenging packaging (ASP) and active-releasing packaging (ARP). Typically, ASP acts as a moisture, carbon dioxide, oxygen, and unpleasant-odor absorber. On the contrary, ARP acts as an antioxidant and antimicrobial and is able to release carbon dioxide and ethanol. The most common AFP technology is focused on the development of labels, sachets, sheets, trays, coating, and pads that can absorb or release gases in a package or headspace, as well as materials containing the active components themselves. [6]. A typical representation of AFP and its main features are shown in Figure 1.

Currently, the active packaging most frequently implemented in the industry involves the use of bioactive molecules which can be directly incorporated into the host matrix, wrapped within an envelope, placed on the host surface as a coating, immobilized on the host surface, or layered into a permeable material and placed alongside the food, allowing the release of volatile agents without direct contact with the food product, simultaneously protecting it against contamination and degradation [7]. Likewise, emerging active packaging technologies focus on the incorporation of natural or synthetic volatile and nonvolatile antimicrobial agents into a polymeric matrix [8]. In this framework, Mallardo et al. reported the customizable fabrication of AFP that incorporated antimicrobial and/or antioxidant agents for the controlled release of substances, or for the absorption of the unwanted substances from both the food and the container’s internal atmosphere, which positively impacted the packaged food [9]. Thus, controlling the active molecule evaporation during processing remains one of the most important technological challenges for the AFP industry. As has been reported in the literature, these drawbacks can be readily overcome by different routes. One of the most promising technological pathways for the fabrication of AFP is based on the association of synthetic or natural polymers with the encapsulation of active molecules (natural volatile essential oils; their main components such as thymol, carvacrol, and triclosan; or synthetic nanoparticles, among others) [10]. As a consequence, the cyclodextrins have been incorporated into food packaging either alone (the so-called “empty” cyclodextrins) or by forming host–guest complexes, named cyclodextrin inclusion complexes (CICs) [11,12,13,14].

**Figure 2:**
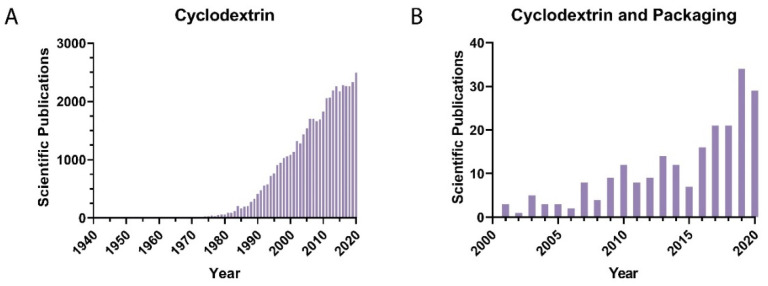
Evolution of scientific publications per year by searching in the Scopus database using the keywords (**A**) “Cyclodextrin” and (**B**) “Cyclodextrin and Packaging”.

The most recent technology advancements from the past few decades have also promoted an increased interest in the research in this area. This can be readily appreciated after a brief bibliometric study. In fact, the number of publications per year in this area demonstrates an exponential growth trend; this can be seen in Figure 2, which shows the evolution of the scientific publications per year found by searching in the Scopus database using the separate keywords “Cyclodextrin” and “Cyclodextrin and Packaging”. From this overview, it can be seen that when only the word “Cyclodextrin” was used, more than 50,000 related papers published in this area were obtained. Additionally, an exponential trend is evident if these results are compared with the previous three decades. Interestingly, when we included “Cyclodextrin and Packaging” in our keywords, we only computed 253 published papers. This result demonstrates the growing interest in developing cutting-edge research related to the use of cyclodextrins and their derivatives in the food packaging industry. It is also worth noting that in both cases, China, the United States, India, and a few countries from Europe such as Italy, France, and Spain are in the top ten countries with the most papers published per year in this research field.

Given the significance of this topic for the agro-food sector, in this review article, we discuss the current status and future prospects regarding the association of cyclodextrins and their derivatives with synthetic or natural polymeric matrices to fabricate cost-efficient, sustainable, and biodegradable active food packaging (AFP). We pay particular attention to the materials and the affordable fabrication technologies available to date, including the trend of nanotechnologies in active food packaging. In addition, we provide a critical view on the possible risks to human health and the updated legislation. Moreover, some patents and commercial products that currently apply AFP technology are also listed and finally, the current and future research challenges which must be addressed are discussed.

## 2. Cyclodextrins (CDs) and Their Derivatives

**Figure 3:**
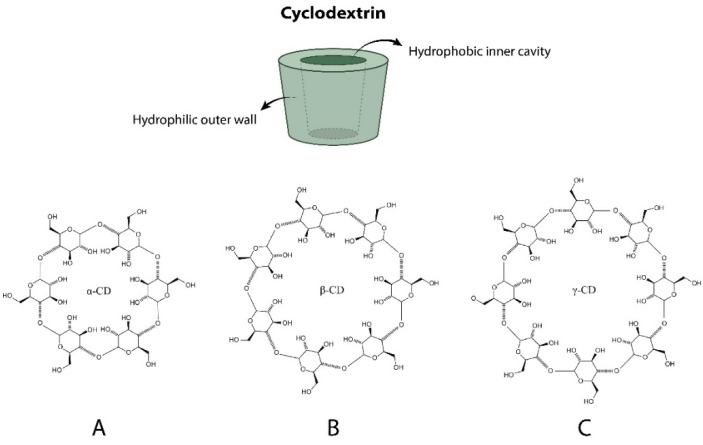
Schematic representation of the typical geometrical shape of native cyclodextrins and their chemical structures for (**A**) α-cyclodextrins, (**B**) β-cyclodextrins, and (**C**) γ-cyclodextrins.

Cyclodextrins were discovered about two centuries ago (1891) by Villiers, who named them cellulosine [15,16,17]. A few years later, Schardinger identified the bacteria capable of degrading starch to produce CDs [18]. Since then, these molecules have been extensively investigated due to their structure and chemical properties. However, it was not until the 1960s and 1970s that Higuchi and Connors proposed a mathematical model to represent the mechanism of the formation of the inclusion complexes used today [19]. CDs are a family of cyclic α-D-glucopyranose oligosaccharides linked by α-1,4 glycosidic bonds that are produced thanks to the biotransformation of starch by microorganisms such as *Bacillus macerans* [20]. The most common cyclodextrins are α-cyclodextrins (six glucose subunits), β-cyclodextrins (seven glucose subunits), and γ-cyclodextrins (eight glucose subunits), recognized as native CDs, with β-CD being the most frequently applied to food [15]. In Figure 3, the schematic representation of the typical geometrical shape of native cyclodextrins and their chemical structures is shown. CDs have a natural origin because they are produced by the enzymatic degradation of starch [15,16,17]. Structurally, CDs have a truncated conical cylinder shape with a nonpolar inner cavity (hydrophobic) and a polar outer surface (hydrophilic) that confers on them the ability to encapsulate hydrophobic substances. The β-CD molecule possesses a complete secondary belt formed by hydrogen bonds, which confers its rather rigid structure and its concomitant lowest water solubility. This hydrogen-bond belt is totally incomplete in the α-CD molecule because one of the glucopyranose units is located at a distorted position. Therefore, only four hydrogen bonds can be fully established, and their solubility in water increases significantly if compared with β-CD. In contrast, the γ-CD possesses a noncoplanar intrinsic structure, which leads to a much more flexible configuration, and for this reason it is the more soluble of the three aforementioned CDs. The thermal and mechanical properties of CDs are also important. The melting points of CDs are relatively high (see Table 1) and their differential scanning calorimetry (DSC) is almost identical, with two heat absorption peaks: the first, at 100 °C, is usually attributed to the water evaporation from the crystals, and the second, located at 250 °C, is attributed to the crystal melting and thermal decomposition. In Table 1, we summarize the main physical and chemical properties of α-, β- and γ-CDs [21,22,23].

Native cyclodextrins are safe to apply in foods; in the USA, they have obtained the GRAS (Generally Recognized as Safe) status as approved by the FDA (US Food and Drug Administration). In Europe, they are considered food additives and labeled as E-457 (α-CDs), E-458 (γ-CDs), and E-459 (β-CDs) [24,25]. The FAO/WHO (Food and Agriculture Organization of the United Nations/World Health Organization) Joint Expert Committee on Food Additives (JECFA) set the maximum advisable level of β-CD in food at 5 mg/kg of body weight per day; however, α-CD and γ-CD do not have an established Acceptable Daily Intake (ADI), due to their benign toxicological profiles [6]. Current regulations suggest that the migration of cyclodextrins from packaging to food does not have harmful effects on consumers if they are part of the packaging; this is why cyclodextrins are recognized as safe additives for use in food [6,26].

The host–guest complexes obtained by the association of CDs with other molecules are defined as cyclodextrin inclusion complexes (CICs), chemical equilibriums governed by the law of mass action, where the CDs are the host molecules [14,27]. The use of inclusion complexes in food packaging contributes to maintaining and releasing active substances in a controlled manner, acting as preservatives and, therefore, reducing the required number of food additives. CICs can also be used on the container’s outer surface to protect against environmental factors [28]; however, it would greatly increase the final price of the active packaging if the internal surface had already been functionalized. Similarly, empty CDs have been used to complex hydrophobic compounds inside packaging, to decrease off-flavors, and to reduce plasticizer migration [29]. Gonzalez-Pereira et al. recently discussed the main applications of CDs in the food industry, including their toxicity impact on human health. However, the active packaging applications involving the use of CDs or CICs were outside the scope of their study [30].

Despite the huge advantages and benefits of the use of cyclodextrins in food packaging formulation, there are, however, a few drawbacks and limitations that are worthy of being taken into account. CDs possess intrinsically limited solubility; therefore, large volumes of water have to be used during any intended formulation. Thus, the reservoir capacity, time, and energy required for heating and cooling may become non-negligible and important factors that would increase the formulation and scale-up cost factors [17]. Likewise, the introduction of novel composites based on CDs and their derivatives in the food packaging industry would be subject to strict regulatory constraints, which would often limit, delay, or hinder its industrial exploitation [15]. Another aspect that must be considered during the fabrication of food packaging composite materials is related to the undesired diffusion of harmful and contaminant substances from the packaging into the food or beverages, which is promoted unintentionally by CDs. Thus, there are necessary issues associated with the detection limit of contaminants in the food [31]. Last but not least, as far as CICs are concerned, the size of the guest molecules must fit within the cyclodextrin’s internal cavity; additionally, the thermodynamic interactions between the CD components (host/guest and solvent) modulate their stability. Furthermore, the stability of the inclusion complex is governed by its formation constant (K_F_) or dissociation constant (K_D_) [32,33].

## 3. Polymers Used in Active Packaging

Food packaging (FP) materials can have different shapes and functions relative to their intrinsic physicochemical properties, and they must guarantee a synergistic balance between their shape and their intended function [1]. Food packages can be customizable depending on their intended use, weight, and shelf life. They might be rigid to produce bottles or trays; flexible to obtain films, wraps, or bags; or semiflexible to produce caps or boxes [34]. The most common food packaging materials used currently in the food industry vary between a wide range of natural and synthetic elements such as metals, paper, glass, and plastics. Nowadays, it is critical to select a suitable material for packaging, depending on the type of food and the final specific functions that the packaging is aimed to fulfill [35]. Although there is a wide range of raw materials that can be used, polymer-based composites for packaging applications remain by far the most suitable material for the food packaging industry. These polymers are classified as biodegradable and nonbiodegradable polymers. We will detail below the most commonly used polymers in the manufacture of films, containers, trays, and other types of packaging [36].

### 3.1. Nonbiodegradable Polymers

The use of nonbiodegradable polymers in the food packaging industry has economical and industrial advantages such as transparency, flexibility, and easy processability. In addition, they are relatively cheap and can be thermally processed and easily manipulated to obtain different forms of packaging. However, their use also exhibits several limitations. According to the 2021–2028 plastic market size, share, and trends analysis report, it is expected that the global plastic market size will increase to more than USD 600 billion by 2021, with an estimated annual growth of 3.4% for the more than 400 million tons of plastics produced per annum worldwide (*Plastic Market Size*, *Growth*, *and Trends Report*, *2021–2028*, s. f.).The packaging end-use has the largest share, representing more than 36% of the market in 2020. Furthermore, the environmental impacts of plastics-based packaging are an extremely important issue. In fact, plastic-based products persist and pollute long after their intended use. The prospects for 2050 are not optimistic, since the oceans could contain more plastic than fish by weight, and the impacts of degraded or intentionally produced microscopic-sized plastics on human health remain unknown. As claimed in a recent European report, about 40% of the total pollution is directly related to packaging waste [37]. These facts are the driving force behind the current challenges to the packaging industry, which pursues efficient, innovative, and biodegradable packaging solutions with environmental advantages [38].

Among the most commonly nonbiodegradable polymers used for the manufacture of food packaging, we can list the following: (i) polypropylene (PP), which is a thermoplastic resin with a high melting point (160 °C), a low density, and a high water-vapor resistance, and thus very suitable for packaging applications that have to withstand high temperatures; (ii) polyethylene-co-vinyl acetate (EVA), which is nontoxic, transparent, flexible, and suitable for the fabrication of thin films composed of copolymerized polyethylene (PE) and amorphous poly(vinyl acetate) (PVA) [39,40], commonly used in refrigerated food packaging because they are highly adhesive, display low vapor permeability, and exhibit heat-sealing features [41,42,43,44]; (iii) high-density polyethylene (HDPE), which is a linear ethylene polymer with small branching, more rigid and less transparent than the low-density variety, and is suitable for the fabrication of film that acts as a barrier against gas and water vapor but is permeable to oxygen and carbon dioxide; (iv) low-density polyethylene (LDPE), which is widely used worldwide due to its low cost, can resist water vapor, but does not retain oxygen, carbon dioxide, and other vapors in food packaging applications [45]; and (v) polyethylene terephthalate (PET), which is a polymer with a high crystalline melting temperature, suitable for bottles and food packaging materials in the food industry, since it exhibits relatively good functional strength and toughness besides its safety and easy processability [46].

Usually, in polymer-based composite packaging fabrication, the bioactive substance is added during the transformation processes. This dictates the type of polymer to be used. The common routes for the fabrication of food packaging include solution–melt mixing, the mold-casting method, thermocasting and -pressing, extrusion blown films, electro spinning, and extrusion or coextrusion technologies. Among these, extrusion stands out as the most frequently used in the industry and involves high temperatures. Consequently, the concomitant properties of the bioactive molecules are compromised due to the high temperatures required to extrude the polymer composite. This is a clear indication that temperature plays a key role in the food industry. Indeed, the temperature at which food is stored can also affect the release rate and shelf life of bioactive components. For these reasons, the direct incorporation of bioactive molecules such as antioxidants or antimicrobials into polymers during the extrusion step has been limited [39,47]. Therefore, at present, innovative, cost-efficient, and eco-friendly alternative pathways are highly appealing to the food packaging industries.

### 3.2. Biodegradable Polymers

As was mentioned above, the increasing awareness of sustainability has motivated the food packaging industry to turn toward the development of innovative technologies that promote the minimization of the environmental impact. This is mainly due to the fact that the products persist and pollute long after their intended use. Thus, the progressive replacement of plastics that are harmful to the environment by biodegradable polymers has become of paramount importance in the last decade. These bioplastics mainly degrade into CO_2_ by the natural enzymatic action of microbes [48,49]. In addition, biodegradable polymers would reduce the pollution problems that are due to plastic packaging waste, since these materials are made mostly from renewable sources that are biodegradable or compostable. So far, they have been classified into four categories based on their chemical composition, origin, and synthesis method, as follows [50]:

- Those obtained directly from biomass (for example, starch, protein, and cellulose).
- Those produced through chemical synthesis from bioderived monomers (for example, polylactic acid (PLA) and biobased polyethylene (PE)).
- Those produced through microbial fermentation (for example, polyhydroxy-alkanoates).
- Those produced through chemical synthesis from bioderived monomers and monomers based on petroleum, butylene polysuccinate (PBS), or polytrimethylene terephthalate (PTT) [51].

Among the biodegradable polymers most commonly used for the manufacture of food packaging, we can list the following.

Polylactic acid (PLA), which is the main biopolymer used in food packaging; its advantages include the fact that it is an excellent thermoplastic and that it is biodegradable and compostable [52,53]. PLA is classified as “Generally Recognize As Safe” (GRAS) by the FDA. It can be rigid or flexible depending on the intended use, being suitable for different applications within the packaging industry; furthermore, its barrier properties are considered similar to those of synthetic polyethylene terephthalate (PET) [54]. Given its useful traits, e.g., that it is nontoxic, noncarcinogenic, biocompatible, hydrophilic, water-soluble, and chemically stable, it is generally mixed with different polymers and used in packaging technologies to produce films, containers, and molding for food and medicine packaging applications.

Natural starch also belongs to the group of the most widely used biopolymers in the food packaging industry and exhibits environmental advantages such as biodegradability and a reduction in the production of carbon dioxide [55]. Starch can act as a thickener, adhesive, or additive. Starch-based films have physical properties similar to those fabricated with synthetic polymers; they are transparent, exhibit no odor or taste, are semi-permeable to carbon dioxide, and are resistant to oxygen transmission. However, in order to fulfill the physical properties required for packaging, starch must be plasticized, that is, subjected to a process that deconstructs the granules, which often results in brittle film materials when dehydrated [56]. The most common polymers used in food packaging applications are summarized in Table 2 [39,48,57]. Table 3 summarizes the oxygen/moisture barrier properties of the polymers most frequently used in the active food packaging industry [58].

Cellulose is another biopolymer widely used in food packaging applications and is considered the most abundant natural polymer; it can be extracted from corn cobs and is eco-friendly and biodegradable [59]. Cellulose by itself is limited to form films; however, cellophane can be produced from it, which has favorable functional properties but is sensitive to moisture [60].

**Figure 4:**
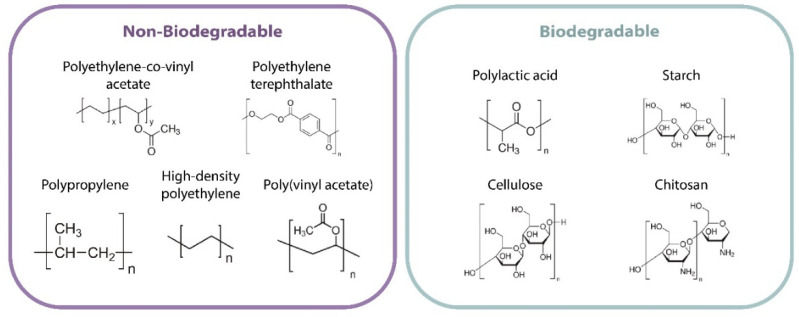
Chemical structures of the nonbiodegradable and biodegradable polymers most frequently used in the active food packaging industry.

Another type of biopolymer less exploited in the packaging industry is chitosan, which has a linear structure obtained from chitin and is the second most abundant natural polysaccharide. Chitin is converted into chitosan through an enzymatic or chemical deacetylation process. This biopolymer also exhibits antimicrobial properties and can be used either as an antimicrobial agent or substrate. Chitosan films have superior optical, mechanical, and oxygen-barrier properties compared to films derived from other similar polysaccharides. They have been successfully used to extend shelf life and keep food fresh in food packaging applications [61]. The chemical structures of the polymers most frequently used in the active food packaging industry are shown in Figure 4.

Biopolymers themselves can form continuous structures that can be crystalline or amorphous, and they can act as a barrier against different substances that should be prevented from interacting with food. Additionally, they are be able to preserve food while still being environmentally friendly. Most of the biodegradable polymers described above have been used to produce flexible packaging and fabricate molds, films, and containers to store fruits and vegetables. Furthermore, these materials can also be used to fabricate modified atmospheric storage (MAP) products [48]. In Figure 4, we list the main nonbiodegradable and degradable polymers used in the active food packaging industries [62]. The aforementioned reasons, alongside the principles of a circular economy and sustainability, are the driving force behind the current and future emergent research trends regarding plastics within food packaging technologies. Indeed, related industries must increase their awareness of replacing the use of nonbiodegradable plastic raw materials with biofriendly and biodegradable ones. Thus, future efforts should be focused on the development of eco-friendly and compostable materials.

## 4. Incorporation of Active Substances in Polymeric Matrix Composites for Active Packaging Applications

The physicochemical properties and the functionality of the biopolymers must be adapted to the market, consumer needs, and the type of food to be packed. This has been commonly carried out through the use of different routes including but not limited to chemical modifications and associations with other substances or molecules, plasticizers, or cyclodextrins [50,55]. The formulation of modern biodegradable packaging must fulfill specific physicochemical requirements. These materials must be capable of forming a mold with sufficient continuity and cohesion in order to protect or pack a product with tailored tensile strength (TS) and elongation (E), but also an adequate oxygen permeability and water vapor transmission rate (WVTR) [63,64]. According to the most recent literature, the current and future research trends in plastics are focused on the development of eco-friendly and compostable materials [63,64].

As was mentioned above, one of the most promising technological pathways for the fabrication of active food packaging is based on the fortification of synthetic or natural polymers with nano/micro capsules containing active molecules, preferably of a natural source. However, it is still challenging to preserve the properties associated with packaging materials, such as the color, odor, antimicrobial or antioxidant activity, and permeability. Various technologies have been used so far to incorporate active substances into a polymeric matrix via chemical encapsulation. In fact, classical technologies used in the food packaging industry are also being used or considered for use in the efficient development of innovative active packaging. Common extrusion and coextrusion, solution–melt mixing, the mold-casting method, thermocasting and -pressing, extrusion-blown films, and electrospinning technologies, to mention just a few, are highly suitable approaches for active food packaging applications [1,35,65,66,67,68]. In a typical procedure, the active molecules can be directly incorporated into the polymeric matrix, wrapped within an envelope, applied to the surface as a coating, immobilized on the polymer surface, or layered into a permeable material and placed alongside the food. Any of these routes must face several drawbacks during its processing. The temperature is one of the most important parameters that must be taken into account. As a result, most of the packaging used in the food industry is produced at high temperatures, between 100 °C and 200 °C, making it difficult to incorporate active substances. Such temperatures are responsible for a molecule’s volatilization and material degradation and other considerable losses of its physical and chemical properties. For example, Suppakul et al. reported that the association of basil essential oil within low-density polyethylene (LDPE) films resulted in very little antimicrobial activity in cheese samples due to the volatilization of the active ingredient during extrusion processing [46]. Similarly, Boonruang et al. recently reported the antifungal properties of polymer composites based on the association of thymol and (R)-(-)-carvone within poly(lactic acid) (PLA). Although they reported acceptable antifungal efficiency, the authors also highlighted that thermal processing within the 80–150 °C range of temperatures resulted in the loss of antifungal properties [3,69]. In addition, Chen et al. reported the advantages of encapsulating antimicrobial agents such as citral (CI) and trans-cinnamaldehyde (TC) into packaging film composites, to minimize the losses with the temperature increment [70]. Since these molecules are easily degraded by the temperatures commonly used in extrusion processing, the encapsulation of these active molecules prevented their degradation via the temperature increase and preserved their bioactive effectiveness during and after the extrusion process. They also highlighted that this route would not only offer an improvement in the thermal sensitivity of such compounds but would also be able to control their release rate.

To solve the temperature and processing drawbacks, the fabrication of tailored, encapsulated polymer-based composites has been widely studied and seems to be one of the most promising and industrially scalable routes so far [4]. Additionally, in line with the EU’s recent research and innovation policy, Food 2030, which aimed to make our food systems ready for the future, linking multiple sectors such as packaging, waste, and recycling, following a bioeconomy strategy, the food packaging industry is also making an effort to replace the use of nonbiodegradable plastic raw materials with biofriendly and biodegradable ones. Indeed, future research challenges will be focused on the production of cost-efficient, eco-friendly, and industrially scalable materials based on the association of biodegradable polymers with molecular encapsulation technologies. In this context, the fabrication of cyclodextrin inclusion complexes (CICs) has been widely used in the literature [11]. These complexes can be applied in a coating layer, in which they are suspended over the polymeric mold to avoid the agglomerations that would limit its functionality [7,71]. To date, various research groups have already demonstrated the development of biodegradable packaging with positive results in terms of its functional characteristics and activity. For example, Friné et al. recently reported the fabrication of an antimicrobial PLA biodegradable packaging material by incorporating thymol and carvacrol inclusion complexes into β-cyclodextrin through an injection technique. These customized receptacles showed an appropriate functional characteristic, as they were able to inhibit *Alternaria alternata* growth [41]. Similarly, Yang et al. demonstrated that starch-based coatings that incorporated natamycin inclusion complexes in methyl-β-cyclodextrin lengthened the shelf life of tomatoes, significantly inhibiting the *Botrytis cinerea* activity [42]. Other interesting examples can be found in literature for when the active ingredient must be released in a controlled way, as in the case of antimicrobial substances, antioxidants, or fragrances [72]. In such composite materials, a miscible polymer with adequate kinetics must be achieved, because a very slow release will not often produce the desired effect. In this circumstance, hydrophobic polymers such as polylactic acid, cellulose, or chitosan are most frequently used to stimulate the release of active substances [28]. On the other hand, masterbatch (MB) has been used to improve the compatibility of some inclusion complexes with less-hydrophilic polymers, to obtain films with enhanced mechanical properties [73]. These facts make it clear that the current and future research efforts into incorporating cyclodextrins are focused on facilitating the biodegradability of packaging materials. In particular, the inclusion complex route is very promising, because it generates some weak points in the structure that facilitates the degradation of the material. This effect would be highly enhanced if the packaging were made of biopolymers [74].

### 4.1. Cyclodextrins in Food Packaging Technologies

The development of active food packaging with cyclodextrins is highly appealing nowadays, mainly due to their truncated conical cylinder shape, with its nonpolar inner cavity and polar outer surface [17]. This configuration makes them especially suitable for the encapsulation of hydrophobic substances, protecting them from volatilization, oxidation, and overheating and simultaneously improving their solubility. Furthermore, they are also highly biocompatible, eco-friendly, and not harmful to human health [14]. Cyclodextrins and their host–guest-complex derivatives such as cyclodextrin inclusion complexes (CICs) are among the most commonly used encapsulation approaches for the development of active food packaging [12,13,34]. As was mentioned above, this technology allows the protection and extension of the shelf life of a variety of foods but also offers a positive environmental impact due to its biodegradable nature [6,30,75]. When CDs are associated with biodegradable-polymer-based hosts, their potential applications increase and the packaging becomes entirely eco-friendly and biodegradable [76,77].

CDs have been incorporated into active food packaging through different routes: (i) alone, the so-called “empty cyclodextrins” that are commonly used to retain the hydrophobic substances released inside the package; (ii) as cyclodextrin inclusion complexes (CICs), which can keep the active substance in the inner cavity and control its release to extend the product’s shelf life without losses in its quality, safety, or organoleptic parameters, but are also used to trap molecules on the outer surface of the package, providing protection against environmental factors; and (iii) CDs can form themselves - supramolecular polymer assemblies. In fact, the intrinsic structure of CDs allows the synthesis and design of CD-based polymers with specific functions and assemblies [78]. This route is suitable to improve the properties and the activity of different active substances [27,79].

#### 4.1.1. Empty Cyclodextrins

**Figure 5:**
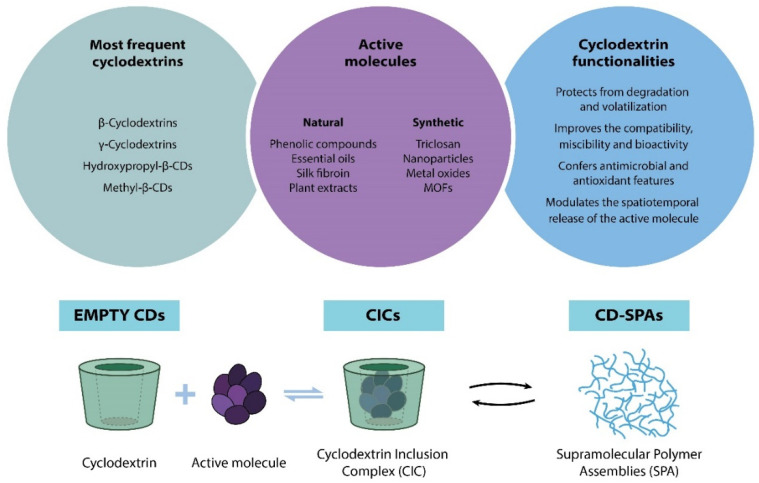
Schematic representation of the cyclodextrin inclusion complex (CIC) formation. The most frequent CICs, the active molecules, and the main CD functionalities for active packaging applications (AFP) are also listed.

CDs can be introduced in empty form into food packaging. Their main function involves trapping or encapsulating undesirable volatile compounds such as hydrophobic molecules that are present either inside or outside the packaging, but they are also used to prevent taste/aroma losses from the packaging and to slow volatile substances’ migration into and out of the packaging [3,6,11,15,24,30,80]. Recently, this technology has been applied for capturing flavors and eliminating unpleasant odors, such as those generated by irradiated meat. An interesting work by Shin et al. reported the versatility of empty CDs in food packaging applications for sulfur-based odor elimination. They reported the use of an electrospun fiber mat based on low-density polyethylene (LDPE) and triacetyl-β-cyclodextrin (TA-β-CD) to eliminate three sulfur odor compounds: (i) dimethyl disulfide (DMDS), up to 90%; (ii) dimethyl sulfide (DMS); and (iii) carbon disulfide (CDS) [29]. Similarly, López de Dicastillo et al. reported the fabrication of polyvinyl alcohol-free standing films doped with β-CD in order to reduce milk cholesterol. They reported an important cholesterol reduction and showed that most of the cholesterol molecules were trapped on the packaging walls used during storage [81]. The same group also demonstrated, in a separate work, the immobilization of β-cyclodextrin in an ethylene–vinyl alcohol copolymer (EVOH) by extrusion with glycerol. Such composite films were flexible and transparent, with a maximum load of β-CD of 30%, and showed increased barrier properties against water vapor, oxygen (O_2_), and carbon dioxide (CO_2_) when compared with those of pure EVOH. These permeability enhancements were ascribed to the presence of discontinuities and hydrophobic cavities within the matrix provided by the β-CD inclusion [82]. In this case, empty CDs applied to plastic containers during the heat-sealing process would also help to eliminate the smell of decomposition derived from this process [11]. Figure 5 shows a typical schematic representation of an empty cyclodextrin accompanied by the main CD functionalities for active packaging applications (AFP).

#### 4.1.2. Active Molecule Encapsulation and Cyclodextrin Inclusion Complex for Active Food Packaging Applications

Encapsulation in food packaging is defined as the process of trapping an active molecule within another macromolecule, producing customized micro- and macroparticles that release their contents during prolonged periods in a controlled way [83]. These substances can be active molecules or not, responsible for conferring the active food packaging characteristics of the material, such as: (i) protecting the active molecule from degradation, volatilization, and undesirable interactions with packaging materials; (ii) improving the compatibility, miscibility, and synergy between the packaging polymer and the active molecule; (iii) confering the antimicrobial/antioxidant and bioactivity enhancement; and (iv) modulating the spatiotemporal control over the release of the active molecule, in order to extend the shelf life and reduce the changes in the sensory properties of foods [20,84].

The chemical complex obtained from the association of CDs with a target molecule is commonly named a cyclodextrin inclusion complex (CIC) in the literature [31]. Due to their versatility, CD inclusion complexes are one of the most promising formulation routes in the active food packaging industry [11,17,80,85]. A schematic representation of the cyclodextrin inclusion complex (CIC) formation by a simple combination of cyclodextrin with a target active compound is shown in Figure 5.

In recent years, several investigations have focused on the use of CICs to stabilize active substances, aiming to confer on the material its antioxidant, antimicrobial, and bioactive properties for food packaging applications. These complexes have also been applied to produce tailored packages as food preservatives [76,80,86]. In such materials, the release rate is of paramount importance and depends on: (i) the final concentration of the active substance within the polymer; (ii) the affinity for the cyclodextrin; (iii) the diffusion coefficient in the polymer; (iv) the partition coefficient between the polymer and the cyclodextrin, and between the polymer and the packaged product; and (iv) the temperature and time [28,34].

Among the CDs most frequently used to obtain inclusion complexes for active food packaging applications are the native β-CD and the modified hydroxypropylated and methylated β-CDs. These are highly efficient at encapsulating and protecting aromatic and/or heterocyclic molecules compared to other classes of cyclodextrins [87,88,89]. In the case of essential oils (EOs) and their active ingredients, such as thymol, carvacrol, linalool, and cinnamaldehyde, the encapsulation step is essential to reducing volatilization or degradation losses during manufacturing or storage. The encapsulation also helps to improve their compatibility and miscibility with biopolymers by increasing their solubility, and it promotes the reduction of the organoleptic impact on food products caused by their strong odor or taste [90,91]. EOs have been widely used in food packaging to extend the shelf life of a variety of foods [92]. Various methods have been used to incorporate them into polymeric molds. Polymer-blend solution stands as the most commonly used so far. They have also been used, directly or via chemical encapsulation, as a coating for containers and for the fabrication of bags [8]. As revealed through an interesting work by Lin et al., gelatin nanofibers containing thyme essential oil were fabricated to form inclusion complexes of β-cyclodextrin in ε-polylysine (TCPN) [93]. These fibers were obtained via ionic gelation, and they exhibited excellent antimicrobial activity against *Campylobacter jejuni* in chicken samples with no adverse effect on the color, texture, and sensory properties. In the same way, Wen et al. reported the incorporation of a β-CD inclusion complex with cinnamon EO into polylactic acid (PLA) nanofibers fabricated by electrospinning [89]. They produced two series of samples with and without EO encapsulation and demonstrated the extended shelf life of pork when they encapsulated the cinnamon EO. Similarly, Santos et al. reported the fabrication of inclusion complexes of carvacrol with HP-β-CD and β-CD. They reported that these CICs showed improved antimicrobial activity against *Escherichia coli K12* and *Salmonella enterica* serovar Typhimurum LT2 [94]. They also demonstrate that a greater antimicrobial capacity was obtained for the encapsulated carvacrol when compared with the nonencapsulated carvacrol. In the same way, Velazquez-Contreras et al. evidenced that PLA packaging containing 5% β-CD-thymol or 5% β-CD-carvacrol extended the shelf life of berries during a storage period of 21 days at 4 °C, revealing a good sensory score and the higher inhibition of yeasts and molds [53].

It is worth mentioning that both carvacrol and thymol remain as the most frequently used natural active components in the food industry. They are naturally present in oregano and thyme EOs and exhibit a high antimicrobial activity. However, both possess a very low solubility in water, which limits their application in food science. To summarize, the most relevant studies regarding the use of cyclodextrin inclusion complexes (CICs) in active food packaging applications are listed in Table 4. It is worth noting that, according to a recent bibliometric study regarding the distribution of the biological applications of cyclodextrins, a significant share (around 20%) of these items concern the antimicrobial activity, just behind the antioxidant activity [79].

#### 4.1.3. Cyclodextrin and Their Derivatives for the Formation of Supramolecular Polymer Assemblies

Another approach to improving the properties and the activity of different active substances in food packaging research is focused on the formulation of CD–polymer supramolecular assemblies. Indeed, the intrinsic structure of CDs allows the synthesis of supramolecular polymeric assemblies based on the association of CDs with monomers or polymers to produce novel composites with tailored structures, properties, and functionalities [78]. So far, CDs and their derivatives had been used to synthesize several linear polymers, polyrotaxanes, rings, helices, and brushes, among others [16,80,109,110,111].

The principal synthesis methods for the formation of such supramolecular assemblies are:

(i) ring-opening polymerization, (ii) Cu-catalyzed azide–alkyne cycloaddition reaction, (iii) atom transfer radical polymerization, and (iv) reversible addition–fragmentation transfer polymerization [112].

It is worth mentioning that the synthesis of polymers with CDs dramatically improves their intrinsic properties, and they can be readily customizable for their intended application. An interesting work was recently published by Zou et al. They reported the fabrication of mechanically reinforced composite films based on the association of high-amylose corn starch/konjac glucomannan (HCS/KGM) with β-cyclodextrin (β-CD), for the development of degradable active packaging materials. They demonstrated that the β-CD was systematically segregated from the polymer chains to form crystals. This promoted a more compact HCS/ KGM film. These materials were transparent and exhibited a reduced moisture formation, with an improved water vapor permeability compared with the native films [113]. Similarly, Huang et al. reported the fabrication of a novel food packaging material based on molecularly imprinted polymers (MIP), using β-CD as a monomer. They combined allyl isothiocyanate MIP (AITC-MIP), chitosan (CS), β-CD, and toluene diisocyanate (TDI) as crosslinkers for beef preservation. These composite coating films exhibited tailored antimicrobial properties and promoted the extended shelf life of the beef, evidenced by the reduction of the muscle deterioration compared with the native films [114]. Using a similar approach, Joo et al. reported the fabrication of biodegradable free-standing films via a cold-casting process based on poly(lactic acid) (PLA) and β-CDs at different loads (up to 30 wt.%). Surprisingly, they claimed that the PLA and β-CDs were immiscible. This effect increased as the CD content increased. They reported that the cyclodextrin tended to form agglomerates at higher loads, promoting a poorer adhesion between both phases. This incompatibility was responsible for the deterioration of the mechanical and oxygen/water-barrier capacity of the PLA. They also demonstrated that a possible route to overcome these undesired effects consisted of the addition of CDs at 30 wt.% in the form of a masterbatch [73]. It should also be noted that the inclusion complex immobilized in a polymeric matrix by physical entrapment is considered a CD–polymer even though the CD units are not linked by covalent bonds [111].

## 5. Methods of Incorporating CDs in Polymer-Based Active Food Packaging

**Figure 6:**
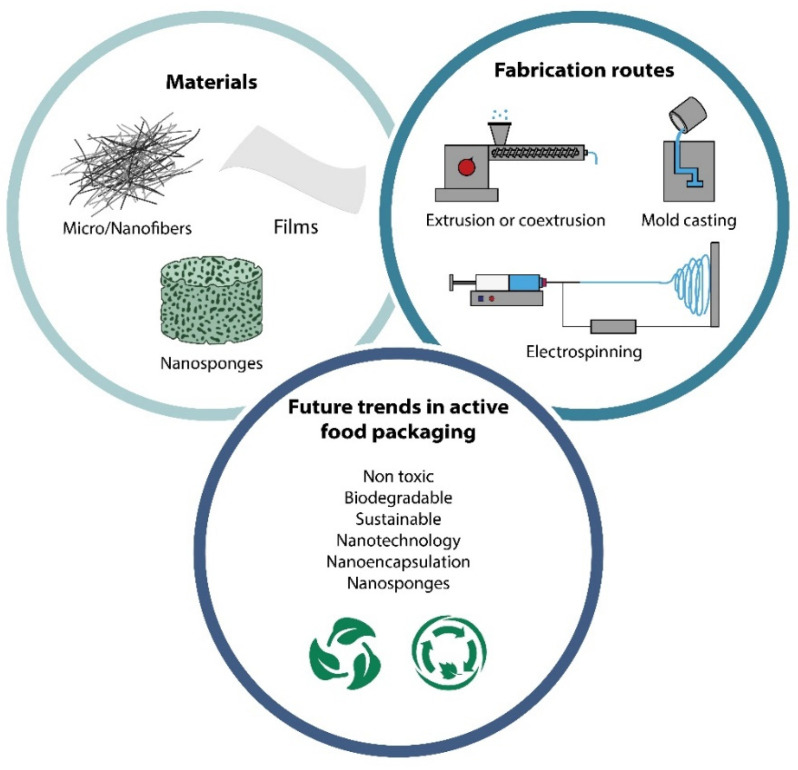
Cyclodextrins and polymers in active food packaging applications: materials and routes for incorporating CDs in polymers.

As was mentioned above, the most promising technological pathways to associating CDs and their derivatives with polymers for the fabrication of active food packaging include extrusion or coextrusion, coating, spraying, casting, solution–melt mixing, the mold casting method, layer-by-layer deposition, thermocasting and -pressing, adsorption, extrusion blown films, and electrospinning, to mention just a few [1,65,66,67] (Figure 6). These techniques exhibit technological advantages and disadvantages depending on the intended use, and they have been used to fabricate different types of active food packaging [68,86,115]. In the next section, we will describe in detail the most promising technologies.

### 5.1. Electrospun Micro-/Nanofibers and Mats

Electrospinning is one of the most appealing technologies for the incorporation of CDs within micro-/nanofibers and mats. This low-cost technique allows the transformation of a given viscoelastic fluid into fibrous membranes under the influence of an electric field and was observed for the first time more than a century ago [116,117]. The final performance of these fibrous materials is influenced by the physicochemical properties of the precursor solution, the experimental variables used during the electrospinning, and the environmental conditions. These factors modulate the resulting membrane structure and its concomitant physicochemical properties. The versatility of this technique lies in the fact that it not only allows the control of the dimensions and diameters of the microparticles and fibers but also permits the modulation of the morphology and texture of their surfaces, with structures ranging from smooth fibers to rough, porous, and hollow structures. The experimental setup of the classical electrospinning technique is quite simple and consists of atomizing a conductive solution by applying a strong electric field. The stabilization of the electrospray process occurs when the electrostatic forces exceed the surface tension of the liquid. Most applications focus on the stable regimen or cone-type jet, which turns out to be the simplest and most reliable method for the production of microparticles, fibers, and mats. It is worth mentioning that the experimental electrospinning setup can be readily tailored for industrial purposes as a pilot line production tool that provides all of the capabilities needed to develop the continuous and customizable manufacture of products, making this technology ideal for the fabrication of scalable pre-production volumes of high-quality tailored materials. To date, different experimental setup configurations exist in the market. These range from typical electrospinning (solution electrospinning) to other customizable setups, such as gas jet electrospinning, magnetic-field-assisted electrospinning, conjugate electrospinning, and centrifugal electrospinning [118,119,120].

As far as food packaging and electrospinning are concerned, the conductive polymeric solution precursor can be made from different polymers such as PVA, PLA, PGA, PLGA PP, HDPE, chitosan, cellulose, gelatin, and zein [68,121]. Moreover, this technology has been successfully applied to melted polymers, polymer solutions, and molecule–nanoparticle mixtures [122,123,124]. Owing to its feasibility and versatility, the electrospinning system solution has been commonly employed in the development of food packaging materials. It provides a high loading capacity and greater stability for the bioactive molecules without the temperature drawback of other techniques, promoting the sustained and controlled release of the embedded molecules [125]. It is also worth noting that, at present, there exist controversies about electrospinning-based materials in food packaging applications. Indeed, there are critical concerns due to the loss of fibers that can penetrate the food; however, this can be avoided by fusing the fibers onto a base substrate that forms a barrier between the food and the fibers. Typically, electrospinning-based materials exhibit low barrier rates against oxygen and water vapor due to their highly porous matrix, which in turn is a critical problem. Aytac et al. used this technique to produce electrospun nanofibers containing thymol inclusion complexes in γ-cyclodextrin (TCICs), with improved antimicrobial activity as demonstrated through the decreased bacterial count in meat stored for 5 days at 4 °C. This effect was not observed in the other nanofibers studied [12]. More recently, the same group reported the development of electrospun fiber mats for potential application as packaging materials [126]. They reported different fibers made of cellulose nanocrystals (CNCs), zein (protein), and starch combined with either pure thyme oil, citric acid, and nisin or their complexed forms as cyclodextrin inclusion complexes (CICs). These composite materials were highly responsive to relative humidity (RH) and were antimicrobial against *E. coli*, *L. innocua*, and *A. fumigatus* [126]. Recently, Sharif et al. demonstrated the antibacterial potential of electrospun fiber mats based on the association of cuminaldehyde (CUM) with the hydroxypropyl-β-cyclodextrin (HP-βCD) inclusion complex. Their antibacterial activity against *Escherichia coli* and *Staphylococcus aureus* confirmed the suitability of this method for the development of films for active packaging applications. It is worth mentioning that they also studied the inclusion complex formation and stability via computational molecular docking [127]. Similarly, Li et al. reported the fabrication of a novel food packaging material based on electrospun nanofibers using a combination of soy lecithin phospholipids, poly(ethylene oxide), and the cinnamaldehyde (CA)/hydroxypropyl-β-cyclodextrin (HP-β-CD) inclusion complex. The phospholipids acted as a surfactant and substantially reduced the surface tension of the precursor solution, which led to a dramatic decrease in the polydispersity of the fibers. These nanofibers exhibited significant antibacterial activity against *Listeria monocytogenes* on fresh-cut cucumber, without altering their sensory properties [128]. Another recently published work by Narayan et al. reported the use of electrospinning to fabricate antibacterial polymeric fiber mats for active food packaging applications. Their formulation was based on the association of poly(vinyl alcohol) (PVA) with caffeic acid (CA)/β-CD and γ-CD inclusion complexes. Its antibacterial activity was demonstrated against the Gram-positive bacteria of *E-coli* and Gram-negative bacteria of *S. aureus* [129]. The versatility of the combination of electrospinning with CD encapsulation has been widely discussed in the recent literature [68].

### 5.2. Nanosponges

An interesting type of supramolecular polymeric material based on cyclodextrins has been recently labeled as cyclodextrin-based nanosponge (CD-NS). This supramolecular assembly is based on cross-linked cyclodextrin polymers nanostructured into a three-dimensional network, and it is considered a new type of biocompatible cross-linked polymer which possesses particular properties and advantages in terms of its biocompatibility, greater protection of encapsulated compounds, higher loading capacity, better solubility, and provision of an efficient method for controlling the release of molecules [104,130]. Depending on the precursors, the nanosponges can be completely nontoxic, and they are able to form stable formulations over a wide range of pH values and temperatures [130,131]. CD-NSs are highly porous nanoparticles characterized by a crystalline or amorphous structure, spherical shape, and swelling properties. They can be formulated indistinctly from any cyclodextrins and their derivatives and can be cross-linked with different molecules such as di-aldehydes, epoxides, epichlorohydrin, and diacyl chlorides, depending on the intended application [12,132]. These nanostructures have been widely used in the pharmaceutical, cosmetic, food, and environmental industries, targeting drug-delivery applications. To date, it has been demonstrated that they are very suitable within the food industry for the encapsulation of antimicrobial agents in packaging materials [24,76,130]. In this context, Simionato et al. recently reported the encapsulation of cinnamon essential oil with α-CD and β-CD nanosponges, demonstrating their ability to encapsulate higher amounts of EOs [104]. They also claimed an improved bacteriostatic effect against *B. thermosphacta*, *L. monocytogenes*, and *E. coli*. Similarly, Silva et al. reported the incorporation of coriander essential oil in CD nanosponges and proved their ability to provide a controlled EO release. They reported an improved bacterial growth inhibition for those materials fabricated from β-CD-derived NSs [130].

Despite the various examples that can be found in the literature, many authors agree on the fact that most of the nanosponge’s potential applications within the food packaging industry have not been fully exploited due to the technological challenges that are still present during its manufacturing scale-up. These technological and industrial drawbacks are mostly related to the poor stability of the active compounds during processing and their uncontrolled molecular release from the packaging material [30,110,130]. These facts constitute the current driving forces behind active food packaging research.

### 5.3. Nanotechnology and Cyclodextrins in Active Food Packaging Applications

Nanotechnology is one of the most appealing and modern transdisciplinary research areas for the formulation of customized materials for a wide range of potential applications in different industrial sectors [133,134,135]. This discipline is more and more present in our day-to-day life and deals with the formulation of cost-efficient, innovative, and well-performing materials and devices within the specific scale-range of atoms and molecules. By definition, nanomaterials are those in which at least one of their constituents are within the range of 1 to 100 nanometers (1 nanometer is defined as one billionth of a meter). At this scale, these materials exhibit novel, enhanced, and tunable physicochemical and biological properties, which can be exploited in many industrial applications. Most nanoparticles exhibit a very high specific surface area and thus have a large potential for trapping or releasing active or bioactive substances and molecules. In this context, the food packaging industry also represents an important niche for the development of modern and competitive nanomaterials. To date, many authors have already reported interesting findings regarding the development of multifunctional materials for food packaging [66,88,136]. Many of these research studies have been focused on the development of smart and active packaging [133,136,137]. One of the most promising routes is also related to the encapsulation, or nanoencapsulation, of specific active/bioactive nanometric particles. In this context, cyclodextrins can adopt a variety of nanometric forms beside their intrinsic ability to act as capping or reducing agents for metallic nanoparticles. CDs can also form stable nanoparticles/fibers or nanomicelles, which can be subsequently utilized for plenty of applications, including active food packaging [138]. An interesting work in this regard has been reported by Lin et al., who detailed different nanoencapsulation strategies for natural compounds based on liposomes and chitosan nanogels [139]. Nanomaterials could play an important role within the food packaging materials industry when a combination of specific active molecules is needed to control the capture or release of molecules, to confer antimicrobial/antioxidant properties, or to extend a food’s shelf life, among other things [133]. Concerning surface microbial food contamination, it is important to highlight that some nanomaterials, such as nanocarbon (carbon nanotubes, graphene, and other nanofullerene derivatives); metallic/magnetic nanoparticles, such as silver, gold, and zinc; and several nano-oxides, such as zinc, magnesium, and titanium (ZnO, MgO, and TiO) exhibit effective and cost-efficient antimicrobial effects against several different bacteria, as well as against various strains of fungi, algae, and certain viruses [134,140,141,142,143]. This represents a huge advantage compared with other natural/synthetic antimicrobial agents that are capable of inhibiting only specific organisms. Due to the nanoscale range of these particles, they are able to disrupt the barriers of lipopolysaccharides and proteins and pierce into the outer and inner membranes of the cells. However, many of the potential applications of nanomaterials in food science are still under debate due to the contradictory results reported regarding the potential risks and cytotoxic effects on human cells [141,144]. As far as cyclodextrins and nanotechnology are concerned, different examples can readily be found in the literature which demonstrate the effectiveness of combining both elements [24,80,145]. A recent study by Kathuria et al. demonstrated the feasibility of producing nanoporous structures with a high surface area, tunable pore size, and selective molecule sorption. They fabricated a nanocrystalline structure using γ-cyclodextrin (γ-CD) and potassium ions by a vapor diffusion process. Such materials, which are commonly known as nano metal–organic frameworks (nano-MOFs), proved to have the ability to encapsulate ethanol through host–guest interactions. This is crucial in the development of active packaging [146]. Another interesting approach is the fabrication of functional nanosponges based on CDs and essential oils, such as those reported by the group of Silva et al. [130]. Very recently, Adeli et al. demonstrated that the application of a simple bioactive coating based on edible gelatin/hydroxypropyl-β-cyclodextrin (HP-βCD) enriched with a nanoemulsion of mustard essential oil extracted from mustard (*Brassica juncea*) seeds inhibited lipid oxidation and significantly delayed the microbial contamination of turkey meat, extending its shelf life substantially [147]. Furthermore, nanofibers based on the association of natural/synthetic polymers and natural essential oils, synthetic antimicrobial components, or nanomaterials seem to be a highly promising route for the fabrication of active packaging [67,68]. Moreover, nanocellulose and biopolymeric silk fibroin nanoparticles (SFNPs) have great potential for the development of innovative food packaging [57,133,148]. SFNPs are particularly interesting due to their unique combination of intrinsic mechanical and biological properties such as biocompatibility and biodegradability [149,150]. However, concerning their industrial processing, the main drawback of these regenerated particles lies in the fact that they are almost insoluble spontaneously in water and in most common organic solvents. This is why their association with cyclodextrin polysaccharides would enhance their potential applications. So far, silk fibroin nanoparticles have been largely used as a carrier for a wide range of bioactive molecules. Additionally, SFNP can act as a natural enzyme immobilizer. This has been crucial in debittering naringin-containing packaging juices [149]. Finally, all these aforementioned materials are the most outstanding precursors for food packaging applications (Figure 6).

## 6. Toxicity and Legislation of Cyclodextrins in Polymer-Based Active Food Packaging

The pioneers on the biological effects, toxicity, and risk assessment of dextrin-derivative-based materials in animals were Pringsheim and French, thanks to their works published almost a century ago [11,151]. In fact, the first trials of Pringsheim and coworkers carried out by the direct administration of cyclodextrins demonstrated their nontoxic nature and their potential for use as a source of energy for diabetics. These results were based on the fact that they were unable to measure significant increases in the sugar levels in the animals’ urine. However, a few years later, French and coworkers reported that, depending on the dose and the chosen dextrin derivative used, the result could be totally different, with a negative impact on the biological response of the animals. Thus, it is important to note the extreme complexing ability of cyclodextrin when interacting with other molecules and biological systems. The hydrophilic CD groups can penetrate, with considerable difficulty, the lipophilic membranes and form complex crystallizations in some organs such as kidneys and eye corneas. Even the lipophilic derivative methylated β-cyclodextrin does not readily permeate lipophilic membranes [17]. However, several studies demonstrated their inertness, attributed to their similarity to other inert macromolecules such as starch and linear dextrin and their lack of absorption from the gastrointestinal tract. Despite the scarce adverse effects reported, CD’s toxicity, as well as its immunogenicity, remains fairly low. In fact, its use has grown continuously over recent decades, not only in the food packaging industry but also in other key industrial sectors such as the pharmaceutical and chemical industries [15,16,30]. There are still a lot of limitations and challenges regarding the use of CDs in food packaging applications. In fact, it has been demonstrated that a high oral intake of CDs above 200 mg/kg/day causes adverse effects and health issues in the digestive system, such as diarrhea [34]. Today, cyclodextrins and most of their derivatives are considered totally safe and nontoxic in controlled doses. However, their toxicity and associated regulations differ from one country to another. In Asia, the United States, and Europe, the α-, β-, and γ-cyclodextrins are considered safe. The β-cyclodextrins are the only ones with well-established safe consumption doses, due to their innocuous effect. In the United States, CDs are considered a GRAS food additive. In Australia and New Zealand, CDs are considered food. In Europe, CDs have been approved as an additive (E-459).

Concerning the current legislation, an interesting work has recently been published by Petitjean et al. They reported on the legal aspect of using CDs in the food industry worldwide [76]. It is also worth noting that the Scientific Committee on Food (SCF), in a report published in 2016, concluded that, based on the available toxicological database, there is no reason to revise the current ADI of 5 mg/kg bw per day for β-cyclodextrin (E 459) [25]. They did, however, recommend that a few actions must be carried out: (i) including the microbiological specifications for β-cyclodextrin (E 459) and (ii) reducing to the lowest level (according to SCF recommendations) the presence of the carcinogenic trichloroethylene as a residual solvent in β-cyclodextrin (E-459) (SCF, 2002a,b,c,d, as referred to by EFSA Scientific Committee (2005) [37,76]).

As far as the food packaging industry is concerned, the harmful effects and risks for human health associated with the direct consumption of CDs and their derivatives are minimized due to their indirect exposure. In fact, CDs in AFP applications are usually cross-linked within the material used as a package, limiting in this way the direct contamination of the food and the final consumers.

## 7. Commercial Products and Patents

Due to the importance and use of CDs in several industries, a large number of patents have been registered. Table 5. presents the recent patents using CDs in active food packaging. The data were obtained from online databases, which include Scopus, WIPO, Worldwide Espacenet, and Google Patents. The results show that most of the patents are focused on the development of sustainable packaging that extends the shelf life of the product. The patents mostly include films, extrusion, and coatings. The intellectual property of the patents has mainly concentrated on the method of preparation and the application. The inventions contemplate the inclusion of CDs with compounds such as essential oils, fibers, and plastics.

Although several patents have been published so far relating to active food packaging applications, commercially available products are still scarce. Only a few companies from the USA, Japan, Germany, Finland, Spain, France, and South Africa are actually leading the active food packaging market (AFP) [4]. They are mainly commercializing different products, such as moisture absorbers, oxygen scavengers, carbon dioxide emitters, and antimicrobial packaging in the form of films, wraps, trays, pads, sachets, and masterbatches, among others. This lack of commercial AFP products is in total agreement with the bibliometric study and the abovementioned research trends in this area (see Figure 2). This also demonstrates that the topic is still open and extremely important, with high commercial potential.

## 8. Conclusions and Future Trends

The use of cyclodextrins and their inclusion complex derivatives for the development of innovative, nontoxic, biodegradable, sustainable, and cost-efficient food packaging has many advantages. First, it improves the compatibility, miscibility, and synergy between the packaging polymer and the active molecule. Second, it protects the active molecule from degradation, volatilization, and undesirable interactions with the packaging materials. Third, it acts as a vehicle to confer on the packaging the antimicrobial/antioxidant and bioactivity enhancement. For most active molecules, the miscibility is crucial, because most are hydrophobic and almost insoluble spontaneously in water or common organic solvents. This is especially important for those natural or synthetic bioactive molecules intended to confer the antimicrobial or antioxidant characteristics on the active food packaging applications. Fourth, it allows the modulation of the spatiotemporal control over the release of active molecules in order to extend the shelf life and reduce the changes in the sensory properties of foods. For their part, cyclodextrin complexes are stable at high temperatures due to the high thermal degradation of CDs (~280 °C). This is responsible for protecting against the degradation of the active ingredients due to the high temperatures of the packaging production methods. Another advantage is their concomitant biodegradability, which allows the development of new environmentally friendly packaging materials that harmonize with the current sustainable market and commercial exigencies.

The use of cyclodextrins in active food packaging applications needs further study in order to mitigate their environmental and human health impact. Their low toxicity and immunogenicity drive the continuously growing research interest not only in the food packaging industries but also in other key industrial sectors such as the pharmaceutical and chemical industries. According to the latest trends reported in the literature, everything points to a focus in the future food packaging trends on a combination of the classic methods with nanotechnology, nanoencapsulation, or the use of nanosponges. Among the processing techniques, the versatility of electrospinning offers a unique opportunity to design and industrially fabricate tailored polymer/CD-based composite materials for the food packaging industry. So far, CDs have been incorporated into active food packaging as empty cyclodextrins, complexed through cyclodextrin inclusion complex derivatives (CICs), or by the supramolecular polymer assemblies of CDs. According to the literature reviewed, the inclusion complex derivatives stand out as the most appealing and promising route for encapsulating bioactive molecules in active packaging.

As far as the legislation is concerned, despite the marked differences in the current policies applied to different regions such as America, Europe, and Asia, most agree on the safety of using CDs and their derivatives in the food sciences, and even more so in the food packaging industries, due to their indirect contact mechanisms. However, it seems highly appealing to consciously re-examine their impact on the environment and human health due to the extreme complexing ability of cyclodextrin when interacting with other molecules and biological systems.

It is worth mentioning that the patents reported so far in this area are focused on the development of sustainable packaging that extends the shelf life of the product and is produced mainly via film extrusion and coatings. Despite the existence of various patents for active food packaging applications, commercially available products are still scarce. Only a few companies from America, Europe, Africa, and Asia are actually leading the market and commercializing products such as moisture absorbers, oxygen scavengers, carbon dioxide emitters, or antimicrobial packaging in the form of films, wraps, trays, pads, sachets, and masterbatches, among others. This lack of commercial AFP products is in total agreement with the bibliometric study and research trends in this area and can be considered as another demonstration that the development of active food packaging based on biodegradable polymers combined with cyclodextrins is still an open and extremely important issue, with high commercial potential.

Finally, the inclusion of CDs in active packaging would play a crucial role in future trends regarding the implementation of the ultimate packaging. Future development shall focus on the fabrication of cost-efficient and sustainable multifunctional packaging combining the advantages of this CD active packaging (intended to maintain or improve its intrinsic properties and extend the shelf life of the packaged food), boosted by the advantages of smart packaging (intended to monitor the condition of the packaged food and provide safety information to stakeholders such as manufacturers, retailers, and consumers), with the ultimate interest of exploiting sustainable packaging (intended to reduce the environmental impact of packaging waste).

## Figures and Tables

**Table 1:** Main physical and chemical properties of α-, β-, and γ-CDs. Data from [21,22,23].

Properties	α-CDs	β-CDs	γ-CDs
Number of glucose units	6	7	8
Molecular weight (g/mol)	972	1135	1297
Melting point (°C)	275	280	275
Solubility in water at 25 °C (%, *w/v*)	14.5	1.9	23.2
Enthalpy; ΔH (kJ/mol)	32.1	34.7	32.3
Entropy; ΔS (J/°K mol)	57.7	48.9	61.4
Cavity diameter (Å)	4.7–5.3	6.0–6.5	7.5–8.3
External diameter (Å)	14.6	15.4	17.5
Approximate volume of cavity (Å^3^)	174.0	262.0	427.0
Crystal forms (from water)	Hexagonal plates	Monoclinic parallelograms	Quadratic prisms
European trade name as food additives	E-457	E-459	E-458
Solubility in: Tetrahydrofuran, methyl isobutyl ketone, methyl isopropyl ketone, acetone, alcohols	0.0	0.0	0.0
Solubility in propylene glycol	0.5	0.5	0.5
Solubility in pyridine	3.5	3.5	3.5
Solubility in ethyleneglycol	7.0	7.0	7.0
Solubility in N-methylpyrrolidone	14.8	14.8	14.8
Solubility in dimethylformamide	28.3	28.3	28.3
Solubility in dimethylsulfoxide	>41	>41	>41

**Table 2:** Polymers used in food packaging applications.

Origin	Types
Natural and biodegradable	Polysaccharides (starch, cellulose, chitin); proteins (gelatin, casein, silk); polyhydroxy alkanoates (PHA), polylactic acid (PLA)
Natural and nonbiodegradable	Polyamides; polyesteramides; unsaturated polyesters;epoxy and phenolic resins
Synthetic and biodegradable	Aliphatic polyesters (polyglycolic acid (PGA), polycaprolactone (PCL), polybutylene succinate (PBS)); polyvinyl alcohol (PVA); polyalkylene dicarboxylates (polyethylene succinate(PES), polybutynel adipate (PBA)); polyanhydrides

**Table 3:** Summary of the oxygen/moisture barrier properties of polymers. Data from [58].

	Polymer	Oxygen Permeation (cc.mil/m^2^-day-atm)	Water Vapor Permeation (g.mil/m^2^-day-kPa)
**Biodegradable**	PHA	8 (23 °C/85%)	106 (23 °C/50%)
		85 (23 °C/0%)	30 (25 °C/100%)
		230 (25 °C/80%)	26 (37.8 °C/100%)
	PLA	132–590 (23 °C/50% or 0%)	63~342 (23 °C/85%)
	PPC	230	162 (23 °C/90%)
	PLA/Chitosan	72 (25 °C/0%)	319 (37.8 °C/95%)
	PBS	208 (23 °C/50%)	175 (25 °C)
		340 (20 °C/90%)	-
	PCL	1990 (25 °C/0%)	137 (23 °C/48%)
	PBAT	2440 (23 °C/50%)	173 (23 °C/75%)
	PGA	1 (30 °C/80%)	10 (40 °C/90%)
**Non** **Biodegradable**	HDPE	2325 (23 °C/0%)	6 (40 °C/90%)
	PP	2500–3000 (23 °C/0%)	5–10 (40 °C/90%)
	PET	40 (23 °C/0%)	15–20 (37.8 °C/90%)
	PVDC	~1 (23 °C/75%)	2 (38 °C/90%)
	PEF	~18 (25 °C/50%)	~30 (25 °C/90%)
	Bio-PE	2140 (23 °C/0%)	~3 (38 °C/90%)
	Nylon 6	40 (23 °C/0%)	295–310 (37.8 °C/90%)
	Polystyrene	4030 (23 °C/0%)	132 (40 °C/90%)
	EVOH	0.5 (23 °C/0%)	33 (40 °C/90%)

**Table 4:** Inclusion complexes with cyclodextrin and essential oils used in active food packaging applications.

Inclusion Complex	Material	Application	Reference
Mustard essential oil/β-cyclodextrin	Cellulose, sulfate film	Antimicrobial edible films, against *E. coli* and *S. aureus*.	[13]
Thymol/γ-cyclodextrin	Zein, nanofibrous web	Antimicrobial food packaging, inhibiting the growth of *E. coli* and *S. aureus* in meat.	[12]
Eucalyptus/β-cyclodextrin	Zein, ultrafine fibers	Antimicrobial, against *S. aureus* and *L. monocytogenes*.	[95]
Carvacrol/HP-β-cyclodextrin	Chitosan, Film	Antimicrobial packaging for chicken filet.	[96]
Thyme/β-CD ε-polylysine	Gelatine, nanofiber film	Antimicrobial packaging, reduction of the activity against *C. jejuni* in coated chicken.	[93]
Tea tree oil/β-cyclodextrin	Poly(ethylene oxide), nanofiber film	Antimicrobial packaging, antibacterial activity against *E. coli* O157:H7, tested on beef.	[97]
Cinnamon–oregano EO/β-cyclodextrin	Chitosan/ poly(vinyl alcohol), nanofiber film	Antifungal activity against *Botrytis* sp.	[98]
Cinnamon EO/β-cyclodextrin	Poly(vinyl alcohol), nanofiber film	Antimicrobial packaging, antibacterial activity against *E. coli* and *S. aureus* in mushrooms.	[99]
D-Limonene/β-cyclodextrin	Poly(butylene succinate), composite film	Antimicrobial food packaging, antibacterial properties against different bacteria straws.	[9]
Thyme/β-cyclodextrin	Inclusion complex extract	Natural antioxidant and antibrowning activities.	[100]
Curcumin, carvacrol/β-cyclodextrin	Cellulose nanocrystals, film	Antimicrobial food packaging, microbial activity against *B. subtilis.*	[87]
Palmarosa EO/β-cyclodextrin	Polyethylene terephthalate (PET)	Antifungal packaging, extends apple shelf life by slowing *P. expansum* growth.	[101]
Galangal root oil/β-cyclodextrin	Gelatin, nanofibers	Inhibitory effect against *E. coli* O157:H7 in beef.	[102]
Basil and pimenta dioica/β-cyclodextrin	Sachets	Potential to be used as food preservative against *S. aureus*, *E. coli*, *L. monocytogenes*, and *P. aeruginosa*.	[103]
Cinnamon EO/CD-nanosponges	α-nanosponges and β-nanosponges	Antimicrobial activity against foodborne bacteria.	[104]
Oregano EO/(α-CD and γ-CD)	PHBV, film	Higher antimicrobial activity against *S. aureus* and *E. coli.*	[105]
Litsea cubeba EO/β-cyclodextrin	Dandelion polysaccharide, nanofiber	Sustained release and long-lasting antibacterial effect against *S. aureus.*	[106]
Clove EO/β-cyclodextrin	Chitosan/ β-cyclodextrin citrate/ oxidized nanocellulose	Higher activity over Gram-negative bacteria (*E. coli* and *P. aeruginosa*).	[107]
Carvacrol, thymol/β-cyclodextrin	Poly(lactic acid) (PLA)	Microbial inhibition of mesophiles, yeast, molds, and coliforms. Extended the shelf life of raspberries and blackberries.	[53]
Carvacrol, oregano, and cinnamon EOs/β-cyclodextrin	Cardboard box	Reduction in microbial growth of mesophiles, psychrophiles, enterobacteria, yeast, and molds. Extended the shelf life of mandarins.	[108]

**Table 5:** Some patents using CDs in active food packaging.

Patent Title	Application	Description	Reference
Active packaging film based on essential oil/β-cyclodextrin inclusion compound and preparation method for active packaging film	Active packaging film	Beta-cyclodextrin; essential oil with broad-spectrum antibacterial performance; by weight, essential oil/ benexate hydrochloride is 4–24% of the total amount.	[152]
Functional gelatin food packaging film and preparation method	Gelatin food packaging film	Using gelatin as the carboxylated beta-cyclodextrin of main raw material compound and natural active matter.	[153]
Cyclodextrin compositions, articles, and methods	A selectively permeable packaging material	Cyclodextrin inclusion complex and a polymer, the composition obtained with electromagnetic irradiation of a cyclodextrin composition comprising one or more radiation-polymerizable monomers and a cyclodextrin complex, the cyclodextrin complex comprising a cyclodextrin compound, and an olefinic inhibitor comprising a cyclopropane.	[154]
Antibacterial quality-guarantee food packaging bag and preparation method thereof	An antibacterial food packaging	Low-density polyethylene, zinc stearate, monoglyceride, polylactic resin, propylene glycol, dioctyl phthalate, ethoxylated alkylamine, porous hydroxyapatite, medical stone, beta-cyclodextrin, chitosan, lanthanum-loaded zinc oxide, 3–6 parts lanthanum-loaded titanium dioxide, and 0.5–1 part natamycin.	[155]
Degradable packaging film for fruit and vegetables	Packaging film	Based on a polyolefin selected from polyethylene (PE), polypropylene (PE), polystyrene (PS), and ethyl vinyl acetate (EVA) and essential oil antimicrobial agents or said essential agent, microencapsulated in an encapsulating agent selected from the group consisting of cyclodextrin (β- or γ-).	[156]
Clove essential oil contained sterilization plastic wrap and preparation method thereof	Clove essential oil contained sterilization plastic wrap	Clove essential oil, beta-cyclodextrin	[157]
Method for preparing antibacterial food packaging preservation film by doping garlic-oil-beta-cyclodextrin inclusion compound with clove oil	An antibacterial food packaging preservation film	Garlic-oil-beta-cyclodextrin inclusion compound with clove oil	[158]
Environmentally friendly food packaging plastic and preparation method thereof	Environmentally friendly food packaging plastic	25–35 parts epoxy modified hyperbranched poly (beta-cyclodextrin) containing azide and vinyl groups, 8–12 parts vinyl polylactic acid, 4–6 parts (Z)-2-(2-aminothiazole 4yl) 2-pentenoic acid, 1–3 parts coupling agent, 0.5–0.9 part fullerene nano/microfibers, and 0.4–0.6 part initiator.	[159]
Application of hydroxypropyl-beta-cyclodextrin in preparation of antibacterial material, food packaging, and preparation method of food packaging	The food packaging includes, but is not limited to, packaging boxes and bags for packaging edible substances, and packaging bottles for packaging edible substances	Hydroxypropyl-β-cyclodextrin is prepared by enzymatically hydrolyzing starch with *Bacillus*, and the *Bacillus* expresses cyclodextrin glucosyltransferase.	[160]
Food packaging films containing natural antibacterial component	Edible films and, more particularly, a method for preparing a food packaging film with antibacterial activity	Perilla oil and cyclodextrin in the mixed dispersion of high-amylose corn starch and konjac glucomannan to prepare an active film.	[161]
Packaging material	Film or sheet for use in “active” packaging systems, capable of inhibiting the growth of microorganisms on the surface of the food product packaged therein	Encapsulated ethanol and a polymeric component selected from chitosan grafted with polyethylene glycol or cyclodextrin, a mixture of chitosan and polyethylene glycol, and a polymer or mixture of polymers for printable paint applied to the other side of the base layer.	[162]

## Abbreviations

ADI	Acceptable daily intake
AFP	Active food packaging
ARP	Active-releasing packaging
ASP	Active-scavenging packaging
CDs	Cyclodextrins
CD-NSs	Cyclodextrin-based nanosponges
CI	Citral
CICs	Cyclodextrin inclusion complexes
DMDS	Dimethyl disulfide
EOs	Essential oils
EVA	Polyethylene-co-vinyl acetate
FDA	Food and Drug Administration
GRAS	Generally Recognized as Safe
HDPE	High-density polyethylene
JECFA	Joint Expert Committee on Food Additives
LDPE	Low-density polyethylene
MAP	Modified atmospheric storage
MB	Masterbatch
PBS	Butylene polysuccinate
PE	Polyethylene
PET	Polyethylene terephthalate
PLA	Polylactic acid
PP	Polypropylene
PTT	Polytrimethylene terephthalate
PVA	Poly(vinyl acetate)
SCF	Scientific Committee on Food
TA-β-CD	Triacetyl-β-cyclodextrin
TC	Trans-cinnamaldehyde
